# RAGE-Dependent Effect of Exogenous Methylglyoxal Intake on Lung Biomechanics in Mice

**DOI:** 10.3390/nu15010023

**Published:** 2022-12-21

**Authors:** Samiya Al-Robaiy, Alexander Navarrete Santos, Andreas Simm

**Affiliations:** 1 Center for Basic Medical Research (ZMG), University Hospital Halle (Saale), Martin Luther University Halle-Wittenberg, 06108 Halle (Saale), Germany; 2 Department of Cardiac Surgery, Middle German Heart Center, University Hospital Halle (Saale), Martin Luther University Halle-Wittenberg, 06120 Halle (Saale), Germany

**Keywords:** methylglyoxal, lung biomechanics, receptor for advanced glycation end-products, ex vivo ventilation

## Abstract

Methylglyoxal (MG) is a known highly reactive dicarbonyl and precursor to free radicals and advanced glycation end-products (AGEs). It is discussed to be involved in tissue aging and in the pathogenesis of different degenerative diseases. The effect of long-term oral administration of MG, simulating dietary MG intake, on the lung biomechanics of wild type (WT) and receptor for advanced glycation end-products knockout (RAGE-KO) mice was studied using an ex vivo ventilation system starting at the age of 6 months and after feeding for 6 and 12 months with MG. Our results showed that MG was taken up in the circulation and efficiently excreted with urine. The amount of free urinary MG measured after 12 months of feeding was lowered. After 12 months feeding, a significant airway resistance increase accompanied by a decrease of the maximal inspiratory airflow was observed in WT animals. No effect of MG in lung function of RAGE-KO mice could be detected. Despite the evidence that MG entered the systemic circulation, no MG-derived AGE accumulation was detected in the lung lysates in dependency on MG-feeding. Our data indicate that the short-term feeding of MG has little effect in vivo. Only after long-term treatment was MG secretion reduced, leading to tissue impairment.

## 1. Introduction

MG is a highly reactive dicarbonyl compound, endogenously produced as a byproduct of the anaerobic glycolysis and lipid peroxidation [1,2]. It reacts randomly with proteins, lipids, and DNA to form advanced glycation end-products [3,4,5]. Formation of AGEs leads to significant vascular complications and damage to organs such as the heart and kidney. It is associated with several pathologies, including diabetes and neurodegenerative and metabolic aging diseases [6,7,8,9].

Under physiological conditions, the glyoxalase system mainly metabolizes intracellular MG in eukaryotic cells. This system plays a major role in the cellular defense against glycation [10,11,12,13].

The process of glycation to form AGEs can lead to protein modifications resulting in loss of structure and function. This leads to the activation of several inflammatory signaling pathways [2,3]. AGE formation by a reaction between MG and the most susceptible amino acids such as arginine, lysine, and cysteine have been studied under physiological conditions [14].

The extracellular matrix (ECM) is a major target for MG and other dicarbonyls [15,16], as long-lived proteins such as collagens and elastin are very susceptible to non-enzymatic modification due to their slow turnover rates [15,17]. Blood vessels, skin, and tendons are well-known targets for protein glycation [17,18,19]. Due to the formation of crosslinks by glycation, increased stiffness and changes in the binding function of the ECM were reported [20,21].

The receptor for advanced glycation end-products (RAGE) was first described as a signal transduction receptor for AGEs [2,17,22]. In the lung, the expression of RAGE is the highest under normal physiological conditions [23]. At the molecular level, the binding of soluble AGEs and MG-modified albumin to the RAGE-receptor activate complex signaling mechanisms. This leads to the production of pro-inflammatory mediators and causes cell stress by generating ROS, contributing to cellular dysfunction and organ damage [7,24].

Exogenous MG is involved in the flavor and color generation of foods and drinks [12,25] and is detected in manufactured foods and beverages. It was reported that coffee, cake, honey, wine, and beer contain high amounts of MG [26,27,28]. According to the committee of toxicity of chemicals in food, the dietary exposure to MG has been estimated as 1.3–3.9 mg/Kg BW/day in food products [29]. Many studies found controversial results about the physiological relevance of dietary MG sources. Some authors claim that dietary MG reacts with food components before absorption and does not reach the circulation [27,30].

This study aimed to find out if long-term dietary intake of MG, the major precursor of AGEs could reach the lung in the form of free MG or its related modifications through the systemic circulation and impair respiratory capacity in healthy adult mice. In addition, the association between RAGE, the AGE-receptor with the highest expression level in the lung under physiological conditions with exogenous dietary MG, was evaluated in this study.

## 2. Materials and Methods

### 2.1. Study Design

MG was given with drinking water to WT-C57BL/6N mice (Charles River, Sulzfeld, Germany) and to RAGE-KO-C57BL/6N mice (provided by Peter Nawroth; Department of Internal Medicine, Heidelberg University, Heidelberg, Germany).

Before starting the experiment, drinking behavior of the WT and the RAGE-KO mice was evaluated for six weeks and the MG amount was set to 500 mg/kg/day [31]. To ensure the stability of MG, the concentration of MG was monitored for two weeks via high performance liquid chromatography (HPLC). No changes in the concentration of MG were detected over this period.

Data of 10–15 animals from each genotype at the age of 6 months were collected and evaluated to define the baseline biomechanical parameters of mice lungs.

For MG-feeding experiments, 60 mice (50% males and 50% females) from every genotype at the age of 6 months were divided into two groups; thirty animals getting tap drinking water supplemented with MG and 30 controls receiving tap water only. Data of lung biomechanics were collected at two time points, 6 and 12 months after starting MG-feeding.

The animal experiments were approved by the local Commission for Animal Protection of the German state Saxony-Anhalt. Mice were maintained at standard conditions (21 °C and 45–60% humidity, ad libitum access to water and food under a 12 h light/dark cycle 07:00 h/19:00 h CET).

### 2.2. Lung Biomechanics Study with an Ex Vivo Ventilation System

The ex vivo respiratory mechanics were studied using the isolated buffer-perfused mouse lung system (Hugo Sachs Elektronik-Harvard Apparatus, March-Hugstetten, Germany) as described in [32,33]. The mice were anesthetized with intraperitoneal ketamine/xylazine (20 µg/kg body weight). To deliver breaths with the artificial thoracic chamber the trachea was cannulated and a constant positive-pressure ventilation (90 breaths/min) with room air was set. Preparation of the mouse lung was performed as described by [32,33]. Krebs–Henseleit (KHL) buffer (Sigma–Aldrich, Steinheim, Germany), supplemented with 0.1% glucose, 0.3% HEPES, and 2% BSA fraction V (AppliChem, Darmstadt, Germany) was used as a perfusion buffer. It was oxygenated with carbogen to yield a pH of 7.4. Negative-pressure ventilation was performed at 90 breaths/min with regular induction of an augmented breath/4 min, constant minimal pleural pressure (2 cmH_2_O), body weight-adapted tidal volume (7 μL/g) and 1 mL/min perfusate flow at 37 °C with a flow for 90 min. After an equilibration period of 20 min, lung physiological data were recorded for 90 min. At the end of the run, the pulmonary artery pressure was monitored within an increasing perfusate flow rate from 1–2 min/mL in 5 steps for 10 min/step. Lung biomechanics data were recorded by the “Pulmonary Mechanics Data Acquisition Software”.

### 2.3. Determination of MG in Drinking Water and in Urine

In this study, HPLC, according to the protocol described in [34], was used to determine free MG in mice urine or in drinking water. In brief, MG reacted with 4,5-diamino-2,6-dihydroxypyrimidine sulfate (DDP; Sigma–Aldrich) to form highly fluorescent lumazine derivatives at 60 °C for 30 min. Creatinine was added to the mixture as a normalization factor. The reaction mixture was filtered through a 0.45 µm pore membrane (Minisart Syringe filter; Sartorius, Göttingen, Germany) and then separated by a Zorba Eclipse xD13-C18 column of a 1200 series HPLC system. The mobile phase was a mixture of a citric acid buffer pH 6.0 and acetonitrile with a final mobile phase composition of 97% and 3%, respectively. The flow rate was 1.0 mL/min. The fluorescence excitation/emission wavelengths used were 330/500 nm for monitoring MG derivatives, and the photometric detector was fixed at 250 nm for creatinine measurement. Data analysis was done with the chemStation software (Agilent Technologies, Santa Clara, CA, USA).

### 2.4. Determination of Intracellular MG-Derived AGEs Concentration

Lung tissues were homogenized in RIPA Buffer (Cell Signaling, ThermoFisher Scientific, Waltham, MA, USA) containing 1 mM PMFS using the Qiagen Lysis II (Qiagen GmbH, Hilden, Germany). The mixture was vortexed for 5 min at maximum speed and then centrifuged at 10,000× *g* for 20 min. The supernatant was collected, and total protein amount was quantified with the Pierce™ BCA Protein Assay kit (ThermoFisher Scientific, Waltham, MA, USA).

Accumulation of intracellular MG-derived AGEs were evaluated with the OxiSelect^TM^ Methylglyoxal Competitive ELISA kit, (Cell Biolabs, San Diego, CA, USA) following the manufacturer’s instructions. The absorbance was determined using a microplate reader (Infinite M200; Tecan Austria GmbH, Groedig, Austria) at 450 nm. For the positive control, 10 mM MG was added to mouse serum and incubated for five days at 37 °C. The negative control consisted of mouse serum incubated without MG.

### 2.5. In Vitro Simulation of Gastrointestinal MG Digestion

Simulated gastric fluid is an aqueous solution at pH of 3.0 with a chew to a final concentration of 800 µM containing high pepsin and a low trypsin amount (350 μg/mL and 19 μg/mL, respectively). In contrast, simulated intestinal fluid had a pH of 7.0 and contained 143 μg/mL pepsin and 24 µg/mL trypsin [35]. MG amount was measured at 37 °C after 0, 2, 4, and 24 h treatment.

### 2.6. Statistical Analysis and Data Presentations

The data were analyzed using the GraphPad Prism 7 software. Means were statistically compared by two-way ANOVA followed by Sidak’s multiple comparison test. *p* values ≤ 0.05 indicate a significant difference between the groups.

## 3. Results

This study aimed to determine the effect of MG intake on lung biomechanics, mimicking the daily consumption of foods with high free MG amounts.

Because of the high capacity of MG to form AGEs, we monitored the respiratory biomechanics in C57BL/6N mice genotypes of either WT or RAGE-KO. The effect of MG feeding in drinking water was studied at the basal level and after 6 and 12 months of MG oral intake.

### 3.1. The Effect of Long-Term Oral Administration of MG on Lung Biomechanics

**Figure 1:**
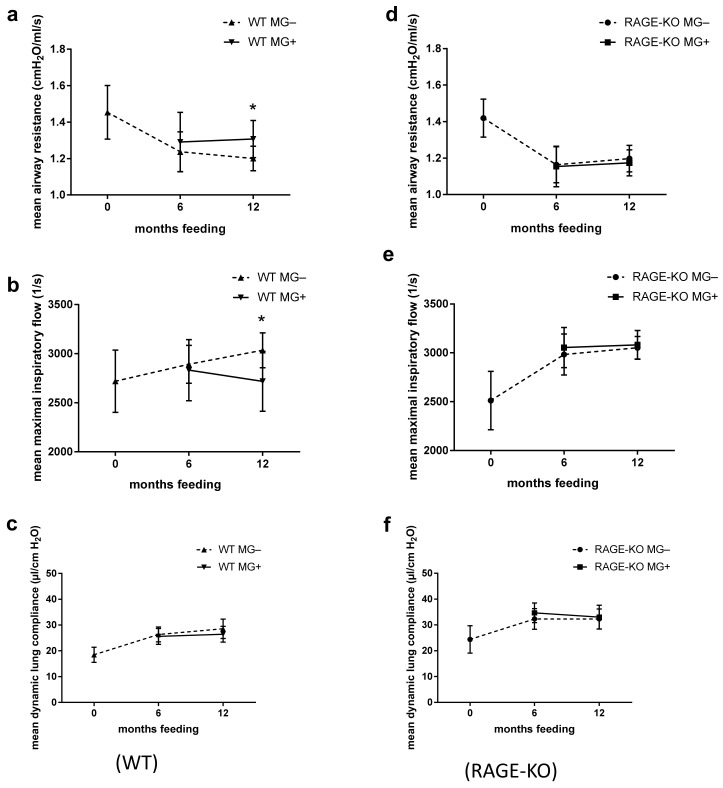
Effect of long-term oral supplementation with Methylglyoxal (MG) on lung biomechanics. Airway resistance, airway maximal inspiratory flow, and dynamic compliance of isolated lungs from (**a**–**c**) wild type (WT) and (**d**–**f**) RAGE knockout (RAGE-KO) mice after 6 and 12 months feeding with MG. Data are shown as mean SD (*n* = 10–15 each group and time point). Means were statistically compared by two-way ANOVA followed by Sidak’s multiple comparison test; * *p* < 0.05.

The ex vivo functional analysis at constant weight-adapted tidal volume for the WT mice showed, after 12 months feeding with MG, significantly higher mean airway resistance with lower maximal inspiratory airflow (Figure 1a,b). Differences were observed only in mice fed for 12 months with MG, while no detectable changes were observed after 6 months MG intake. The dynamic lung compliance did not change over the experimental time (Figure 1c).

In mice lacking the RAGE receptor, the outcome in all of the parameters over the experimental period did not differ between those given MG in drinking water or tap water only (Figure 1d–f).

However, lung dynamic compliance dependency on RAGE was observed in all tested mice regardless of whether they belong to feeding or control mice groups. In lungs of RAGE-KO mice, the dynamic compliance was higher than that of the WT mice over the experimental time (Figure 1c,f).

**Figure 2:**
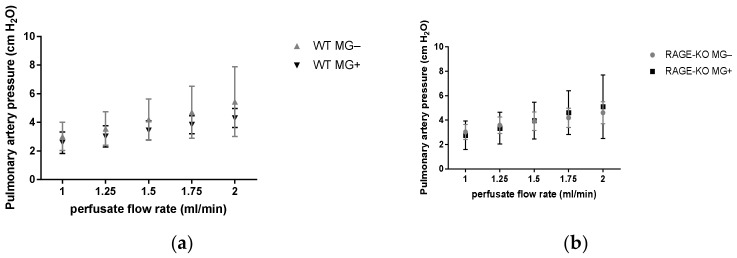
Pulmonary artery pressure measurement in isolated lungs in an increased perfusion flow rate at 1.00, 1.25, 1.50, 1.75, and 2.00 mL/min measured in mice supplemented for 12 months with Methylglyoxal (MG). No significant differences between fed (**a**) wild type (WT) and (**b**) RAGE knockout (RAGE-KO) and controls were observed. Data are shown as mean SD (*n* = 10 each genotype, group, and time point). Means were statistically compared by two-way ANOVA followed by Sidak’s multiple comparison test.

Measurements of the pulmonary artery pressure with gradually increased perfusate flow 1.0, 1.25, 1.5, 1.75, and 2.0 mL/min led to increased values in all the experimental groups. No differences between mice having MG and controls could be detected in the WT as well as RAGE-KO mice (Figure 2).

### 3.2. Detection of MG-Derived AGEs in Mice Lung Lysates

**Figure 3:**
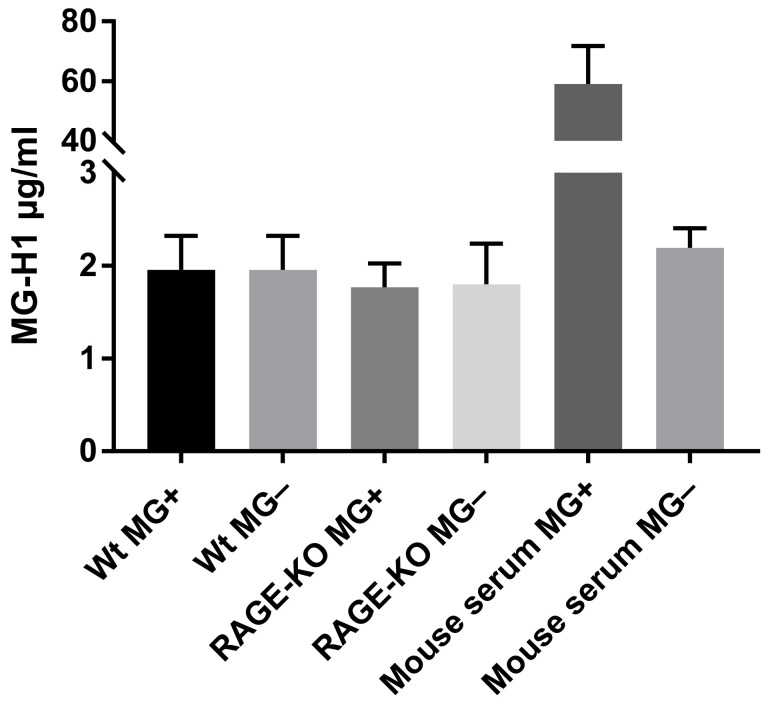
MG-derived AGEs formation in lung lysates after 12 months of feeding with MG or with tap water. Data are shown as mean SD (*n* = 5 in each group). No significant differences within the four experimental groups were determined using one-way ANOVA. The positive control represents mouse serum incubated for 5 days at 37 °C with 10 mM MG in comparison with the negative control, serum without addition of MG.

The hypothesis that changes in lung biomechanical properties in WT mice are due to increased levels of MG-derived AGEs, which bind to the RAGE receptor, activate signaling pathways that results in modification of the lung ECM, was analyzed with ELISA. The detection and quantification of MG-H1 (methyl-glyoxal-hydro imidazolone), the most prevalent MG-derived AGE modification in the lung tissue lysates, did not show any differences due to long-term administration of MG, neither in WT nor in RAGE-KO mice (Figure 3).

### 3.3. Detection of Free MG in Urine after Long-Term Oral Administration of MG

**Figure 4:**
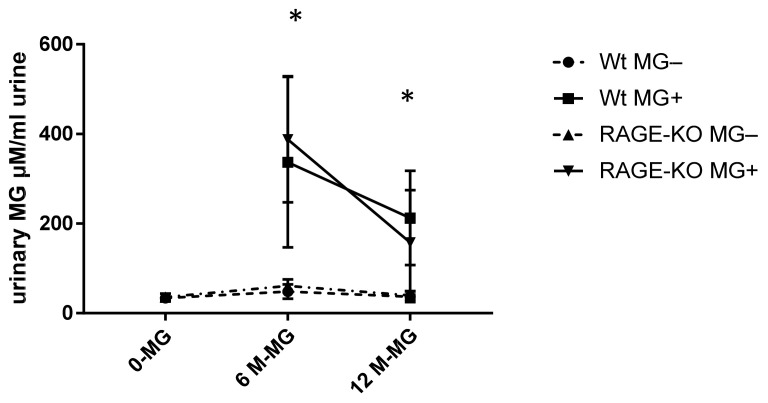
Urinary methylglyoxal (MG) measured with HPLC and normalized on creatinine in urine of wild type (WT) and RAGE knockout (RAGE-KO) mice after supplementation with MG for 6 and 12 months. There was a significant difference between MG amount excreted in fed mice to the controls having tap water; *p* < 0.001. In addition, after 6 months feeding, the excreted MG in urine was significantly higher than after 12 months * *p* < 0.05. Data are shown as mean SD (*n* = 10–12 for each genotype, group, and time point). Means were statistically compared by two-way ANOVA followed by Sidak’s multiple comparison test.

To examine if MG can pass the gastrointestinal tract and enter the systemic circulation, free MG was measured in urine with HPLC. It was shown that a significant amount of MG is excreted with urine of mice fed with MG. However, the rate of excreted MG is highest after six months of feeding and reduced after 12 months of feeding, independent of mice genotype (Figure 4).

### 3.4. In Vitro Simulating Gastrointestinal MG Digestion

**Figure 5:**
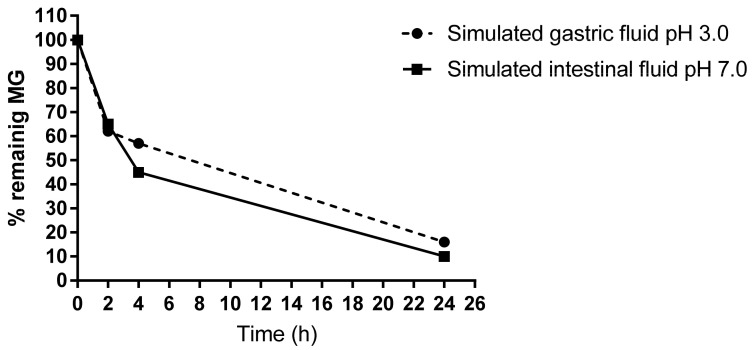
HPLC measurement of free MG remaining in the gastrointestinal digestion mix. The simulated gastric and intestinal fluids were analyzed after an incubation time of 0, 2, 4, and 24 h at 37 °C. About 40% of free MG disappeared after 4 h.

To test the stability of MG, in vitro imitation of gastrointestinal conditions in the presence of food showed that only 40% of MG could be detected after 4 h digestion in the simulated gastric as well as in intestinal fluid. About 10% of MG remains in the reaction fluids after 24 h (Figure 5).

## 4. Discussion

This study aimed to investigate whether dietary free MG can reach the lung through the systemic circulation and impair respiratory capacity. Although MG, a major precursor of AGE formation, was found in high levels in manufactured foods and beverages and RAGE expression is highest in the lung under physiological conditions [23], no in vivo data exist showing biomechanical properties of the lung after long-term administration of MG.

The results proved that oral supplementation of mice with high MG concentration through drinking water mimicking daily intake for a period of one year led to a slight but significant increase in airway resistance and a decrease in maximal inspiratory flow. However, these effects could only be observed in WT mice after 12 months of feeding, whereas in animals lacking the RAGE receptor, no changes in lung function parameters could be detected.

On the other hand, dynamic lung compliance was not affected even after 12 months of MG feeding. The higher compliance measured in the RAGE-KO mice confirms our early results in which increased dynamic compliance was based on worse elastic recoil in the lungs of these mice [33].

Since MG influences respiratory parameters only in WT mice, it can be hypothesized that at least a part of the supplemented MG reacted to AGEs. AGEs/RAGE activation could influence several signaling pathways, leading to the secretion of numerous pro-inflammatory and profibrotic growth factors that can modulate the cell-matrix [15,36]. Many studies showed that long-lived proteins such as collagens and elastin are very susceptible to non-enzymatic modification due to the slow turnover rates [15,16,37].

Fessel et al. studied the association between AGE-formation on collagens and the correlated progressive stiffening of tissues. Evidence was provided that the accumulation of AGEs dramatically affects collagen fibril failure behavior and stress relaxation. They concluded that the MG-induced AGEs could reduce collagen fibril viscoelasticity [17].

To test this in our model, AGE formation in lung tissue lysates was measured after 12 months of feeding with MG by the analysis of Methylglyoxal hydroimidazolone (MG-H1), the most prevalent MG-derived AGE modification found in vivo [37]. Differences for MG-H1 between fed mice and controls were detected neither in WT nor in RAGE-KO mice.

We could not confirm a direct association between AGE-formation and lung respiratory changes from these results. In addition, the pulmonary artery pressure showed no alteration due to MG-feeding. We were not able to detect a stiffening in the pulmonary arteries.

Many studies analyzed the metabolic fate of orally administrated MG. It was reported that only about 20% of the initial MG level remains after the duodenal phase [38]. They suggested that the disappearance in MG was due to interactions of reactive dicarbonyl compounds with the accumulating amino acids during the digestive process. In addition, the effect of dietary MG intake was analyzed in in vitro experiments simulating static and dynamic gastrointestinal digestion processes [30]. It was demonstrated that MG metabolization involves the gastrointestinal tract due to the detoxification of MG in the gastric compartment or the uptake of dicarbonyls bound to amino acids leading to an almost complete metabolization for all the tested concentrations.

In line with this, our in vitro experiments for the gastric and the intestinal digestion reaction mix proved a decrease in free MG to about 60% after 4 h or 90% after 24 h.

In many studies, oral administration of MG does not raise MG-H1 formation in serum or change mRNA expression level of the AGE receptor RAGE [20,24,39]. In addition, no alteration in fasting glucose or insulin resistance could be detected after long-term MG feeding [24,31]. In contrast to the limited effects after oral MG feeding, it was shown that prolonged intraperitoneal administration of MG induced diabetic characteristics associated with microvascular damages and structural modifications [40]. Additionally, in an ex vivo model it was shown that diabetic mice develop structural alterations of the lung and kidneys to the micro and macrovascular endothelium after infusion of AGE-albumin [41].

As MG levels drops by imitation of gastrointestinal digestion, and as we could not detect MG-modified AGEs in lung after one year of MG feeding, it was necessary to determine if MG passed the gastrointestinal tract to the bloodstream. For this purpose, free MG was measured in the urine of the mice. The significant rise in MG excretion after oral administration indicates that at least a part of the fed MG reached the bloodstream. These data are in agreement with the measurements in which a correlation between MG concentration in drinking water and urine was detected without changing plasma MG levels [42].

It was reported that MG could induce significant structural changes in serum albumin [41,43]. MG-modified albumin has a very high nanomolar affinity to bind RAGE, while the well-characterized AGEs such as carboxymethyl lysine (CML) or carboxyethyl lysine (CEL) have weak binding in a micromolar range [37,44].

In many studies with orally administrated MG, increased inflammatory infiltrations and ROS were detected in spite of unchanged RAGE mRNA levels, serum MG and glyoxalase1 values, in addition to unaltered fasting glucose or insulin resistance [20,24,31,39]. The high affinity of MG-modified albumin to bind RAGE could play a role for these results.

The large proportions of free MG excreted with urine do not preclude that a small residual amount could remain in the bloodstream. Yet, MG oral feeding could affect the respiratory biomechanics in WT mice after long-term oral intake. In this study, the proportion of MG remaining in serum was under the detection limit. It was shown that a slight increase could only be detected in serum from mice fed for 18 months with MG [42].

Aging is a condition that favors AGE accumulation. This occurs in addition to increased oxidative stress and less efficient repair processes mainly due to decreased kidney activity regardless of if, the individual has diabetes or not [45]. The reduced excreted MG amounts in urine after 12 months in feeding groups might indicate age-dependent limitations in renal function. 

## 5. Conclusions

Form the results of our study, we suggest that a large amount of daily orally taken MG will be detoxified and metabolized in the gastrointestinal tract. The main free MG that pass the gastrointestinal tract can efficiently be excreted with urine. However, a certain MG amount remains and affects the respiratory biomechanics of the lung. We suggest that these changes might be due to the binding of modified proteins in a nanomolar concentration to RAGE that activate signaling pathways and modulate biomechanical function of the lung after long-term MG oral intake.
